# General Spanish population normative data analysis for the EORTC QLQ-C30 by sex, age, and health condition

**DOI:** 10.1186/s12955-021-01820-x

**Published:** 2021-08-30

**Authors:** Juan Ignacio Arraras, Sandra Nolte, Gregor Liegl, Matthias Rose, Ana Manterola, Jose Juan Illarramendi, Uxue Zarandona, Mikel Rico, Lucia Teiejria, Gemma Asin, Irene Hernandez, Marta Barrado, Ruth Vera, Fabio Efficace, Johannes M. Giesinger, the EORTC Quality of Life Group

**Affiliations:** 1 Oncology Departments, Complejo Hospitalario de Navarra, Irunlarrea 3, 31008 Pamplona, Spain; 2 Charité – Universitätsmedizin Berlin, corporate member of Freie Universität Berlin and Humboldt-Universität zu Berlin, Medical Clinic, Department of Psychosomatic Medicine, Berlin, Germany; 3 Radiotherapeutic Oncology Department, Complejo Hospitalario de Navarra, Irunlarrea 3, 31008 Pamplona, Spain; 4 Medical Oncology Department, Complejo Hospitalario de Navarra, Irunlarrea 3, 31008 Pamplona, Spain; 5 Health Outcomes Research Unit, Italian Group for Adult Hematologic Diseases (GIMEMA) Data Center, Rome, Italy; 6 University Hospital of Psychiatry II, Medical University of Innsbruck, Anichstrasse 35, 6020 Innsbruck, Austria

**Keywords:** EORTC QLQ-C30, Normative values, Spain, General population, Questionnaire, Quality of life

## Abstract

**Purpose:**

General population normative data for the European Organisation for Research and Treatment of Cancer (EORTC) QLQ-C30 questionnaire facilitates interpretation of data assessed from cancer patients. This study aims to present normative data of the general Spanish population.

**Methods/patients:**

Data were obtained from a prior larger study collecting EORTC QLQ-C30 norm data across 15 countries. Data were stratified by sex and age groups (18–39, 40–49, 50–59, 60–69 and > 70 years). Sex and age distribution were weighted according to population distribution statistics. Sex- and age-specific normative values were analysed separately, as were participants with versus those without health conditions. Multiple linear regression was used to estimate the association of each of the EORTC QLQ-C30 scales with the determinants age, sex, sex-by-age interaction term, and health condition.

**Results:**

In total, 1,165 Spanish individuals participated in the study. Differences were found by sex and age. The largest sex-related differences were seen in fatigue, emotional functioning, and global QOL (Quality of Life), favouring men. The largest age differences were seen in emotional functioning, insomnia, and pain, with middle-aged groups having the worst scores. Those > 60 years old scored better than those < 60 years old on all scales except for physical functioning. Participants with no health conditions scored better in all QLQ-C30 domains.

**Conclusions:**

The present study highlights differences in HRQOL between specific sex/age strata and especially between people with and without a health condition in the general Spanish population. These factors must be considered when comparing general population HRQOL data with that of cancer patients.

**Supplementary Information:**

The online version contains supplementary material available at 10.1186/s12955-021-01820-x.

## Background

Health-related quality of Life (HRQOL) is a key outcome in oncology that is widely assessed in clinical studies of patients with cancer [1] and it is now frequently integrated into treatment evaluation in clinical practice [2]. HRQOL is typically assessed with standardised questionnaires whose scores are to be appropriately interpreted to obtain clinically relevant information [3]. The availability of reference data from general population samples improves the interpretability of the data. Population norms are useful in clinical work to assess individual patients’ needs, use as target values for patients, and interpret the results of clinical studies and clinical trials [4, 5].

A true HRQOL baseline assessment is always missing prior to diagnosis and frequently prior to starting treatment in studies of patients with cancer [1, 5, 6], as newly diagnosed patients may already have physical or emotional symptoms. Therefore, reference values from population norms may be useful to substitute missing baseline values.

The European Organisation for Research and Treatment of Cancer (EORTC) Quality of Life Group (QLG) developed the HRQOL core questionnaire, the QLQ-C30, more than 25 years ago [7]. This 30-item instrument is one of the most widely used cancer-specific HRQOL questionnaires [4, 8–10], covering key cancer symptoms and aspects of functional health. More recently, a summary score was developed [11]. This EORTC QLQ-C30 Summary Score was introduced to supplement the detailed 15-scale profile of the QLQ-C30.

Several supplements have been developed to facilitate interpretation of QLQ-C30 scores: a reference values manual for cancer patients that also includes data from the general population [12]; thresholds for clinical interpretation of QLQ-C30 scales [13]; and a definition of minimal important differences (MID) [14]. Additionally, general population norms from the QLQ-C30 have been obtained for specific Northern and Central European countries [5, 6, 15–23] as well as from non-European countries [24, 25]. However, the QLQ-C30’s normative data for countries in Southern Europe – except Croatia [26] – are lacking. Reference HRQOL data from that region may differ from that of other areas due to possible cross-cultural differences [27].

Basic participant characteristics, such as age, sex, and health conditions, may also impact general population HRQOL scores; hence, they should be considered when interpreting HRQOL results [5]. For example, older people constitute the largest group of oncology patients [28], and maintaining HRQOL is a key aim of their treatment [29]. Furthermore, studies indicate men report better functioning and fewer symptoms than women [21, 25], and that the presence and severity of symptoms increase while function declines with age [21]. Furthermore, health conditions, such as chronic pain or diabetes, may also impact the areas measured by the QLQ-C30 [5, 6, 21, 24].

A recent study provided EORTC QLQ-C30 general population normative data pooled from 15 countries: 11 from within the European Union (including Spain) plus Russia, Turkey, Canada, and the United States [30]. Substantial variation in mean QLQ-C30 scores across countries was observed in this study, thereby underscoring the need for country-specific normative values. In this previous publication [30], no country-specific normative values were provided for groups defined by sex, age, and presence of a health condition. Therefore, we aim to report HRQOL normative data for the general Spanish population in this previously collected data set, including age- and sex-specific values, plus values for people with versus those without health conditions.

## Material and methods

### Sampling

The Spanish norm data sample was collected as part of a larger study that was aimed at establishing European general population norm data for the EORTC QLQ-C30 [30]. All Spanish patients from this previous study were included in our analysis. These data were collected in spring 2017 via online panels by GfK SE (www.gfk.com), a large market research institute whose panels are representative for the general population in a given country based on criteria such as age, gender, education, household size, size of the city, and geographical location. As these were online panels, sample representativeness refers to the general population of a given country with internet access. Further details on the data collection are reported elsewhere [30].

The population sample was stratified by sex and age, and included 100 women and 100 men per pre-specified age stratum (18–39, 40–49, 50–59, 60–69 and ≥ 70 years) allowing for sufficient sample sizes per group to establish normative values of age- and sex-specific subgroups. However, stratification resulted in a non-representative age- and sex-distribution; thus, post-hoc weighting of the data was required. Weighting was done according to the sex and age distributions indicated in the United Nations official 2015 population distribution statistics report [31].

Sociodemographic data were collected, which included sex, age, education, marital and employment status, and presence of self-reported health conditions, including cancer, via an online data form. Participants were asked to report only health conditions diagnosed by a doctor by choosing from a list of health conditions or entering additional conditions as free text. Additional conditions were screened by two authors independently, to evaluate whether any could be added to the pre-defined categories in the list provided.

### The EORTC QLQ-C30 questionnaire

The EORTC QLQ-C30 [7] includes 30 items covering five functioning scales (physical, role, social, emotional, and cognitive functioning), nine symptom scales (fatigue, pain, nausea/vomiting, dyspnoea, sleep disturbances, appetite loss, constipation, diarrhoea, and financial difficulties), and a global QOL scale. The questionnaire’s Spanish version has been validated for use in Spain [32]. All questions are answered on a 4-point Likert-type scale, except for two global QOL items using a 7-point scale. The questionnaire scales are scored on a 0–100 metric according to the standard EORTC scoring algorithm [33]. For the functioning scales and the global QoL scale, a higher score indicates better health. For the symptom scales, a higher score indicates a higher level of symptom burden.

The recently introduced QLQ-C30 Summary Score [11] was calculated as the mean of the combined 13 QLQ-C30 scale scores (excluding financial impact and Global QoL). [11]. For this summary score a higher score indicates better health.

### Statistical analyses

Normative values are given as means and standard deviations (SD) separately for women and men in five age groups (18–39, 40–49, 50–59, 60–60, and 70 + years) and in combined sex and age groups. Furthermore, we calculated normative scores for participants with and without health conditions within each group.

As in prior studies [16, 20, 34], we also used multivariable linear regression to estimate the association of each of the QLQ-C30 scales (dependent variable) with age (linear and quadratic term), sex (0 = men, 1 = women), sex-by-age interaction term, and health condition (0 = none, 1 = one or more). Since all participants were 18 or older, we used an age variable by counting the years above 18 to estimate regression coefficients (i.e. participant age minus 18). The regression models predict normative scores for individuals or patient groups based on their sex, age and health conditions more precisely than the normative tables indicate. SPSS version 25.0 was used for all analyses.

## Results

### Participant characteristics

In total, 1,165 Spanish individuals participated in the study. The raw (unweighted) data set included 54.2% men (weighted, 48.6%); the mean age was 54.3 (SD 14.7) years (weighted, 48.1 [SD 16.5] years). The applied weights for the individual participants ranged from 0.36 to 3.52.

In the weighted data, 91.8% of the sample had at least post-compulsory education, 70.9% were married/in a steady relationship, 52.7% were working, and 61.6% presented one or more health condition(s). Detailed sample characteristics are presented in Table 1 and in Supplementary Table S2, where data are presented in Five Age categories.

**Table 1:** Participants’ demographic characteristics (N = 1,165)

		Unweighted data	Weighted data
Sex N (%)	Male	632 (54.2%)	567 (48.6%)
	Female	533 (45.8%)	598 (51.4%)
Age	M (SD)	54.3 (14.7)	48.1 (16.5)
	Median [IQR]	56 [43–66]	48 [34–61]
Age (grouped) N (%)	18–39 years	209 (17.9%)	406 (34.9%)
	40–49 years	213 (18.3%)	227 (19.5%)
	50–59 years	221 (19.0%)	197 (16.9%)
	60–69 years	305 (26.2%)	146 (12.5%)
	≥ 70 years	217 (18.6%)	189 (16.2%)
Education N (%)	Below compulsory education	14 (1.2%)	15 (1.3%)
	Compulsory school	83 (7.2%)	79 (6.8%)
	Some post-compulsory school	132 (11.4%)	117 (10.1%)
	Post-compulsory below university	360 (31.1%)	334 (28.8%)
	University degree (bachelor)	374 (32.3%)	392 (33.9%)
	Postgraduate degree	196 (16.9%)	220 (19.0%)
	Prefer not to answer	6	8
Marital status N (%)	Single/not in a steady relationship	120 (10.3%)	188 (16.2%)
	Married or in a steady relationship	854 (73.6%)	823 (70.9%)
	Separated/divorced/widowed	187 (16.1%)	150 (12.9%)
	Prefer not to answer	4	3
Employment status N (%)	Full-time employed	437 (37.6%)	507 (43.7%)
	Part-time employed	87 (7.5%)	104 (9.0%)
	Homemaker	88 (7.6%)	85 (7.3%)
	Student	14 (1.2%)	38 (3.3%)
	Unemployed	109 (9.4%)	112 (9.7%)
	Retired	352 (30.3%)	245 (21.1%)
	Self-employed	59 (5.1%)	49 (4.3%)
	Other	17 (1.5%)	19 (1.6%)
	Prefer not to answer	2	4
Comorbidity N (%)	None	391 (34.8%)	429 (38.4%)
	One or more	733 (65.2%)	688 (61.6%)
	Chronic pain	252 (22.4%)	239 (21.4%)
	Heart disease	55 (4.9%)	42 (3.7%)
	Cancer	31 (2.8%)	26 (2.3%)
	Depression	110 (9.8%)	113 (10.1%)
	COPD	47 (4.2%)	35 (3.1%)
	Arthritis	103 (9.2%)	96 (8.6%)
	Diabetes	135 (12.0%)	113 (10.1%)
	Asthma	59 (5.2%)	74 (6.6%)
	Anxiety disorder	97 (8.6%)	100 (9.0%)
	Obesity	148 (13.2%)	142 (12.7%)
	Drug/alcohol disorder	4 (0.4%)	6 (0.6%)
	Other	208 (18.5%)	180 (16.1%)
	Prefer not to answer	35	42
	Missing	6	6

### Normative data for the general Spanish population

Table 2 shows the EORTC QLQ-C30 reference values for the general population of Spain. The scores for the global sample in the functional scales ranged between 85.7 and 87.8, except for emotional functioning (77.1). Symptom scores were > 20 points in fatigue, insomnia, and pain. The mean summary score was 84.8. For further details please see Table 2. Floor and ceiling effects for the EORTC QLQ-C30 scales (weighted data) are shown in Table 3.

**Table 2:** EORTC QLQ-C30 reference values for the general population of Spain

	All		18–39 years		40–49 years		50–59 years		60–69 years		≥ 70 years	
	N = 1165		N = 406		N = 227		N = 197		N = 146		N = 189	
	Mean	SD	Mean	SD	Mean	SD	Mean	SD	Mean	SD	Mean	SD
Physical functioning	86.8	16.8	87.1	16.5	87.0	17.9	87.9	15.1	88.9	14.9	83.4	18.7
Role functioning	86.1	21.5	85.6	21.1	84.3	22.5	86.7	21.4	89.5	20.3	86.0	22.0
Emotional Functioning	77.1	22.4	74.7	24.6	73.1	22.7	75.9	21.8	80.9	19.9	85.1	16.8
Cognitive Functioning	85.7	19.4	85.6	21.2	83.5	20.6	85.7	21.1	87.3	16.2	87.2	13.3
Social functioning	87.8	22.5	86.5	24.4	83.9	24.3	88.2	21.0	92.8	17.7	91.4	19.5
Global QOL	66.8	21.5	67.0	21.1	63.0	20.8	67.6	22.4	70.9	20.3	67.3	22.4
Fatigue	23.9	22.7	25.4	23.9	26.1	21.7	25.0	23.1	18.8	21.0	20.4	21.0
Nausea/vomiting	4.9	14.5	7.4	18.2	5.7	14.7	4.0	12.5	2.6	10.3	1.4	7.3
Pain	22.7	24.0	21.9	24.0	26.6	24.8	24.6	24.2	17.6	21.6	21.6	23.8
Dyspnoea	12.4	20.7	13.1	21.1	13.7	21.4	12.4	20.3	10.8	21.4	10.5	18.7
Insomnia	25.2	28.0	26.3	29.1	28.1	27.7	28.3	28.9	21.0	26.0	19.2	25.2
Appetite loss	9.5	19.9	12.7	22.9	9.4	19.0	8.0	17.2	6.4	17.4	6.8	17.5
Constipation	15.3	24.1	16.4	26.0	14.4	22.2	15.3	24.9	14.1	21.3	15.1	23.0
Diarrhoea	7.8	18.1	10.4	20.8	8.9	18.2	7.0	16.9	5.5	13.7	3.7	14.6
Financial problems	9.5	20.7	10.9	21.7	13.4	24.9	10.0	21.8	5.3	15.6	4.5	12.5
Summary score	84.8	15.1	83.5	17.3	83.0	14.7	84.6	14.2	87.9	12.8	87.3	12.3

**Table 3:** Floor and ceiling effects in the EORTC QLQ-C30 scales (weighted data)

	Lowest possible score (0 points) (%)	Highest possible score (100 points) (%)
Physical functioning	0.4	36.9
Role functioning	0.9	61.0
Emotional functioning	1.1	25.2
Cognitive functioning	1.1	50.8
Social functioning	1.5	69.7
Global QOL	0.9	8.5
Fatigue	26.2	1.4
Nausea/vomiting	85.7	0.6
Pain	37.4	2.0
Dyspnoea	69.3	1.1
Insomnia	46.3	4.2
Appetite loss	77.9	1.1
Constipation	64.8	2.9
Diarrhoea	81.1	1.1
Financial problems	79.0	1.7
Summary score	0.3	4.7

### Normative data by sex and age

Table 4 shows descriptive statistics by sex. In the weighted descriptive data, the largest mean differences by sex were fatigue (men 21.6 vs women 26.5 points), emotional functioning (men 79.2 vs women 75.0 points), and global QOL (men 68.4 vs women 65.3 points), with better QOL in men. Mean differences for physical functioning, dyspnoea, financial problems, and summary score were below 1 point (see Tables 4 and 5).

**Table 4:** EORTC QLQ-C30 reference values for women and men in the general population of Spain

	Men												Women											
	Total		18–39 years		40–49 years		50–59 years		60–69 years		≥ 70 years		Total		18–39 years		40–49 years		50–59 years		60–69 years		≥ 70 years	
	N = 567		N = 206		N = 115		N = 98		N = 70		N = 78		N = 598		N = 201		N = 112		N = 99		N = 76		N = 111	
	Mean	SD	Mean	SD	Mean	SD	Mean	SD	Mean	SD	Mean	SD	Mean	SD	Mean	SD	Mean	SD	Mean	SD	Mean	SD	Mean	SD
Physical functioning	86.8	18.2	84.8	19.0	86.0	20.7	89.8	14.9	89.8	15.5	87.0	17.4	86.8	15.4	89.5	13.2	88.1	14.6	85.9	15.2	88.0	14.3	80.9	19.2
Role functioning	84.9	22.7	81.8	23.3	83.0	24.7	86.3	22.4	89.7	19.9	89.7	19.0	87.2	20.3	89.4	17.8	85.6	20.1	87.1	20.5	89.4	20.8	83.3	23.6
Emotional functioning	79.2	21.8	75.6	25.2	76.5	20.8	79.1	19.6	83.0	18.9	89.5	13.1	75.0	22.9	73.9	24.0	69.7	24.0	72.7	23.4	79.0	20.7	82.0	18.4
Cognitive functioning	86.9	19.2	85.9	22.5	84.8	20.2	89.5	16.7	87.8	16.6	89.0	12.0	84.5	19.6	85.4	19.9	82.1	21.1	82.0	24.2	86.9	15.9	86.0	14.0
Social functioning	88.9	21.7	86.4	25.5	85.6	22.2	90.7	18.4	93.7	17.4	94.0	15.1	86.8	23.1	86.5	23.2	82.1	26.3	85.8	23.1	92.0	18.1	89.5	21.9
Global QOL	68.4	20.5	68.2	20.4	64.0	19.4	67.7	22.3	73.3	17.9	72.4	20.7	65.3	22.3	65.7	21.8	62.0	22.2	67.4	22.5	68.8	22.2	63.8	22.9
Fatigue	21.0	21.6	25.1	22.9	23.1	21.0	19.5	19.5	16.4	21.3	13.3	19.4	26.5	23.3	25.8	25.0	29.3	22.1	30.4	25.1	21.0	20.6	25.4	20.7
Nausea/vomiting	5.8	16.8	9.6	22.6	6.8	16.9	3.0	8.5	2.3	10.2	0.7	4.0	4.1	11.9	5.1	11.7	4.5	12.0	5.0	15.4	2.9	10.5	1.8	8.8
Pain	21.8	23.9	25.7	25.7	24.1	24.7	21.9	23.4	16.0	20.7	13.5	18.2	23.5	24.0	18.0	21.4	29.2	24.9	27.3	24.8	19.0	22.4	27.3	25.6
Dyspnoea	12.5	21.3	13.2	21.3	14.9	22.6	12.6	21.6	10.6	20.2	8.8	19.8	12.2	20.1	13.0	20.8	12.3	20.2	12.1	19.0	10.9	22.5	11.7	18.0
Insomnia	24.1	27.1	28.8	30.8	24.8	24.5	23.1	25.4	21.0	25.3	14.2	21.2	26.2	28.7	23.7	27.1	31.5	30.5	33.3	31.3	20.9	26.9	22.7	27.2
Appetite loss	10.6	21.2	17.2	26.4	9.2	18.2	6.3	14.6	5.7	16.7	5.1	16.2	8.5	18.5	8.0	17.6	9.6	19.9	9.7	19.3	7.1	18.1	8.0	18.4
Constipation	14.3	23.3	16.7	26.5	14.9	21.7	12.0	21.9	13.4	21.4	10.5	19.4	16.3	24.8	16.1	25.7	13.9	22.8	18.5	27.3	14.7	21.4	18.3	24.8
Diarrhoea	8.4	18.6	12.9	24.0	8.6	16.0	5.1	12.9	6.4	14.1	2.3	11.4	7.3	17.5	7.8	16.4	9.3	20.3	8.8	20.0	4.7	13.3	4.7	16.4
Financial problems	9.9	21.5	13.2	24.7	11.4	22.6	9.0	22.0	5.2	14.8	4.3	11.2	9.1	20.0	8.5	17.8	15.4	27.1	10.9	21.7	5.3	16.4	4.7	13.4
Summary score	85.2	16.1	81.9	19.7	83.8	15.8	87.1	12.4	88.6	12.3	90.8	10.0	84.3	14.1	85.2	14.2	82.2	13.5	82.2	15.5	87.2	13.2	84.8	13.2

**Table 5:** EORTC QLQ-C30 reference values for the general population of Spain by age, sex, and health condition

	Male																							
	18–39 years				40–49 years				50–59 years				60–69 years				70 + years				Total			
	One or more health conditions N = 94		No health condition N = 99		One or more health conditions N = 75		No health condition N = 34		One or more health conditions N = 59		No health condition N = 33		One or more health conditions N = 43		No health condition N = 25		One or more health conditions N = 59		No health condition N = 17		One or more health conditions N = 329		No health condition N = 209	
	Mean	SD	Mean	SD	Mean	SD	Mean	SD	Mean	SD	Mean	SD	Mean	SD	Mean	SD	Mean	SD	Mean	SD	Mean	SD	Mean	SD
Physical functioning	81.8	15.9	88.6	21.6	81.4	21.9	98.1	4.3	87.2	16.4	96.3	6.9	85.3	17.6	96.8	7.6	84.2	18.9	96.2	6.6	83.6	18.3	93.0	16.1
Role functioning	74.9	21.3	89.9	22.5	77.0	25.4	98.9	4.2	80.3	25.6	97.8	6.9	84.9	23.2	97.1	9.6	87.1	20.7	98.7	6.6	79.8	23.6	94.2	16.7
Emotional functioning	67.4	25.9	83.6	23.0	72.5	23.7	85.8	10.3	73.9	20.3	88.2	15.6	78.4	20.3	90.6	13.6	88.0	14.1	94.2	8.2	74.8	23.0	86.4	18.5
Cognitive functioning	78.5	22.7	91.5	21.6	79.9	22.4	94.1	10.1	86.1	19.2	95.2	9.4	84.9	18.2	93.0	12.1	87.1	12.3	96.2	7.2	82.6	20.1	93.1	16.5
Social functioning	78.8	27.9	93.5	22.0	79.7	24.7	98.9	6.0	86.8	21.3	97.4	9.9	90.2	20.7	99.0	8.1	92.8	16.4	100.0	0.0	84.4	23.9	96.2	16.3
Global QOL	58.4	21.1	77.8	15.6	56.4	17.5	80.6	11.8	60.9	22.3	79.4	18.8	66.8	18.4	84.1	11.1	68.0	20.9	86.5	13.3	61.2	20.6	80.0	15.1
Fatigue	32.8	21.4	17.4	22.5	29.1	21.7	9.0	12.6	26.0	20.8	7.9	10.3	23.0	23.6	5.6	11.4	16.2	21.0	4.7	9.6	26.5	22.2	12.0	18.2
Nausea/vomiting	14.8	25.3	5.7	20.4	9.3	19.8	1.6	6.6	4.5	10.3	0.4	2.7	2.7	11.2	1.7	8.8	0.6	3.1	0.0	0.0	7.6	18.3	3.2	14.8
Pain	37.0	23.2	14.5	23.0	31.9	25.5	6.5	10.2	28.1	24.0	9.2	12.7	21.8	22.8	6.3	12.3	15.5	19.4	5.8	10.6	28.4	24.3	10.6	18.2
Dyspnoea	16.4	19.4	10.4	23.3	20.6	25.1	3.2	10.0	16.9	24.9	4.4	11.4	15.4	23.1	3.4	11.8	11.0	21.9	2.6	9.2	16.3	22.8	6.8	18.1
Insomnia	38.5	31.0	21.9	29.4	30.4	25.6	12.9	16.5	27.4	27.2	14.9	20.1	28.5	26.6	8.7	17.0	16.3	22.7	7.7	14.5	29.4	28.0	16.5	24.4
Appetite loss	24.0	27.3	10.3	23.2	12.3	20.7	2.2	8.3	7.0	14.9	3.5	10.4	8.8	20.4	0.5	4.1	6.4	18.2	0.0	0.0	13.2	22.6	5.8	17.4
Constipation	25.0	29.2	9.5	22.3	19.1	23.2	5.4	12.4	16.4	25.6	5.3	12.3	17.1	23.6	7.7	16.5	13.3	21.2	2.6	9.2	19.0	25.4	7.4	18.1
Diarrhoea	18.7	26.1	8.6	22.0	10.3	17.5	6.5	13.4	6.5	14.5	2.6	9.1	8.5	15.4	3.4	11.8	2.7	12.6	0.0	0.0	10.4	19.8	6.0	17.2
Financial problems	20.9	26.0	6.5	22.5	16.2	26.1	1.1	6.0	13.4	26.7	0.9	5.4	7.7	17.3	1.4	9.0	5.7	12.6	0.0	0.0	14.1	23.8	3.6	16.3
Summary score	74.9	17.5	88.4	20.3	79.0	17.2	94.5	5.4	83.2	13.6	94.4	6.1	84.5	12.8	95.3	7.9	89.0	10.6	97.1	3.8	81.1	15.9	91.9	15.0

	**Female**																							
	**18-39 years**				**40-49 years**				**50-59 years**				**60-69 years**				**70+ years**				**Total**			
	**One or more health conditions N = 88**		**No health condition N = 102**		**One or more health conditions N = 69**		**No health condition N = 40**		**One or more health conditions N = 65**		**No health condition N = 32**		**One or more health conditions N = 49**		**No health condition N = 24**		**One or more health conditions N = 88**		**No health condition N = 21**		**One or more health conditions N = 359**		**No health condition N = 220**	
	**Mean**	**SD**	**Mean**	**SD**	**Mean**	**SD**	**Mean**	**SD**	**Mean**	**SD**	**Mean**	**SD**	**Mean**	**SD**	**Mean**	**SD**	**Mean**	**SD**	**Mean**	**SD**	**Mean**	**SD**	**Mean**	**SD**
Physical functioning	84.2	15.5	93.5	9.4	83.2	16.1	96.6	5.5	83.1	16.0	91.5	12.0	84.3	16.0	94.4	6.9	77.4	19.9	95.4	5.9	82.1	17.1	94.0	8.8
Role functioning	80.6	20.9	96.3	10.9	79.1	22.2	97.4	7.2	83.3	22.7	94.9	13.1	84.2	24.1	98.6	6.2	79.7	24.7	100.0	0.0	81.1	22.8	96.9	9.8
Emotional functioning	65.1	25.6	81.9	20.2	64.4	24.4	80.6	17.1	68.6	24.9	81.9	16.0	74.8	22.7	87.5	14.0	80.6	18.3	87.3	18.9	70.7	24.0	82.8	18.4
Cognitive functioning	79.6	20.4	89.4	19.0	77.4	23.0	91.5	12.0	76.2	27.4	92.6	10.1	83.1	17.5	93.5	9.2	85.0	14.0	91.2	10.2	80.3	20.9	90.9	15.1
Social functioning	76.1	26.7	95.0	15.9	75.4	28.9	94.9	11.6	81.7	26.3	94.4	12.0	88.4	21.5	99.1	3.9	86.9	24.0	99.1	3.8	81.3	26.2	95.7	12.9
Global QOL	53.4	21.9	76.2	16.1	52.6	19.5	78.8	15.9	61.5	22.5	80.3	16.9	61.4	22.6	82.4	13.4	59.0	22.1	82.5	16.4	57.2	21.9	78.6	16.0
Fatigue	36.6	27.2	17.3	19.8	38.5	20.9	12.8	12.9	37.0	26.1	17.3	17.9	27.5	21.7	9.0	11.6	28.4	20.9	11.1	10.4	33.8	24.0	15.0	17.0
Nausea/vomiting	9.7	15.3	1.6	5.7	6.7	14.2	0.9	5.3	6.7	18.1	1.9	7.8	4.6	12.9	0.0	0.0	2.3	9.9	0.0	0.0	6.1	14.4	1.2	5.4
Pain	30.7	23.7	8.4	12.6	38.1	24.9	13.2	15.8	33.1	25.3	15.3	20.1	26.9	23.6	4.6	9.5	33.1	25.4	5.3	9.7	32.6	24.7	9.6	14.4
Dyspnoea	23.2	23.6	5.5	14.5	18.9	22.6	1.7	7.4	16.2	20.9	3.7	10.6	16.4	26.2	0.9	5.6	14.3	19.0	0.0	0.0	18.0	22.4	3.5	11.5
Insomnia	35.9	26.9	14.9	23.7	41.8	31.4	13.7	18.3	37.0	32.4	24.1	26.0	27.9	29.5	9.3	15.2	25.3	26.8	10.5	24.9	33.6	29.7	15.0	22.7
Appetite loss	15.1	22.8	2.7	9.2	12.9	23.2	4.3	11.3	13.0	22.1	3.7	10.6	10.0	21.4	1.9	7.8	9.7	20.1	1.8	7.6	12.3	21.9	3.0	9.5
Constipation	20.5	27.1	9.9	20.1	14.9	22.7	12.8	23.7	20.8	30.4	13.9	20.2	16.0	23.1	12.0	18.2	19.4	25.4	12.3	19.9	18.6	26.0	11.5	20.5
Diarrhoea	14.5	20.9	2.2	8.3	12.4	23.8	3.4	10.2	8.8	20.2	6.5	13.4	5.9	15.1	2.8	9.4	5.9	18.3	0.0	0.0	9.8	20.3	2.9	9.4
Financial problems	13.2	21.0	3.9	13.2	22.9	31.4	3.4	10.2	15.3	24.4	1.9	11.1	8.2	19.9	0.0	0.0	5.9	14.9	0.0	0.0	13.0	23.3	2.7	10.9
Summary score	76.9	14.6	91.8	10.1	76.6	13.3	92.2	6.6	78.5	17.1	89.9	7.8	83.0	14.6	94.8	4.2	82.4	13.5	94.8	5.4	79.3	14.8	92.2	8.4

	Total																							
	18–39 years				40–49 years				50–59 years				60–69 years				70 + years				Total			
	One or more health conditions N = 182		No health condition N = 201		One or more health conditions N = 144		No health condition N = 74		One or more health conditions N = 124		No health condition N = 66		One or more health conditions N = 92		No health condition N = 49		One or more health conditions N = 146		No health condition N = 38		One or more health conditions N = 688		No health condition N = 429	
	Mean	SD	Mean	SD	Mean	SD	Mean	SD	Mean	SD	Mean	SD	Mean	SD	Mean	SD	Mean	SD	Mean	SD	Mean	SD	Mean	SD
Physical functioning	83.0	15.7	91.1	16.7	82.2	19.3	97.3	5.0	85.0	16.3	93.9	10.0	84.8	16.7	95.7	7.3	80.1	19.7	95.8	6.2	82.8	17.6	93.5	12.9
Role functioning	77.7	21.3	93.2	17.8	78.0	23.8	98.1	6.0	81.9	24.1	96.4	10.5	84.6	23.6	97.8	8.0	82.7	23.4	99.4	4.4	80.5	23.2	95.6	13.6
Emotional functioning	66.2	25.7	82.8	21.6	68.6	24.3	82.9	14.6	71.1	22.9	85.1	16.0	76.5	21.6	89.1	13.7	83.5	17.1	90.4	15.3	72.7	23.6	84.6	18.5
Cognitive functioning	79.0	21.6	90.5	20.3	78.7	22.6	92.7	11.2	80.9	24.3	93.9	9.8	83.9	17.8	93.3	10.7	85.9	13.4	93.4	9.2	81.4	20.5	92.0	15.8
Social functioning	77.5	27.3	94.2	19.1	77.6	26.8	96.7	9.6	84.1	24.1	95.9	11.0	89.2	21.1	99.1	6.3	89.3	21.4	99.5	2.8	82.8	25.1	96.0	14.6
Global QOL	56.0	21.6	77.0	15.8	54.6	18.5	79.7	14.1	61.2	22.3	79.8	17.8	63.9	20.8	83.2	12.2	62.6	22.0	84.3	15.0	59.1	21.4	79.3	15.6
Fatigue	34.7	24.4	17.3	21.1	33.6	21.8	11.1	12.8	31.8	24.2	12.5	15.2	25.4	22.6	7.3	11.5	23.5	21.7	8.2	10.5	30.3	23.4	13.5	17.6
Nausea/vomiting	12.4	21.1	3.6	15.0	8.1	17.3	1.2	5.9	5.6	14.9	1.1	5.8	3.7	12.1	0.9	6.2	1.6	7.9	0.0	0.0	6.8	16.4	2.2	11.1
Pain	33.9	23.6	11.4	18.7	34.8	25.3	10.1	13.9	30.7	24.7	12.2	16.9	24.5	23.2	5.5	10.9	26.1	24.7	5.5	10.0	30.6	24.6	10.1	16.3
Dyspnoea	19.7	21.8	7.9	19.4	19.8	23.9	2.4	8.7	16.5	22.8	4.1	11.0	15.9	24.7	2.2	9.3	13.0	20.2	1.2	6.2	17.2	22.6	5.1	15.1
Insomnia	37.3	29.0	18.3	26.8	35.9	29.0	13.3	17.4	32.4	30.3	19.4	23.5	28.1	28.0	9.0	16.0	21.7	25.5	9.2	20.7	31.6	29.0	15.7	23.5
Appetite loss	19.7	25.6	6.4	17.9	12.6	21.8	3.3	10.0	10.1	19.1	3.6	10.4	9.5	20.8	1.2	6.2	8.4	19.3	1.0	5.7	12.7	22.2	4.4	14.0
Constipation	22.8	28.3	9.7	21.2	17.1	23.0	9.4	19.6	18.7	28.2	9.5	17.1	16.5	23.2	9.8	17.3	16.9	23.9	7.9	16.5	18.8	25.7	9.5	19.4
Diarrhoea	16.7	23.7	5.4	16.8	11.3	20.7	4.8	11.8	7.7	17.7	4.5	11.5	7.1	15.2	3.1	10.6	4.6	16.3	0.0	0.0	10.1	20.0	4.4	13.8
Financial problems	17.2	23.9	5.2	18.4	19.4	28.8	2.3	8.6	14.4	25.4	1.4	8.7	8.0	18.7	0.7	6.4	5.8	14.0	0.0	0.0	13.5	23.5	3.1	13.8
Summary score	75.9	16.2	90.1	16.0	77.8	15.4	93.2	6.2	80.7	15.6	92.2	7.3	83.7	13.8	95.1	6.3	85.1	12.8	95.8	4.8	80.2	15.3	92.1	12.0

The largest pairwise mean differences between age groups were observed for emotional functioning (age 40–49 years: 73.1 points vs age 70 + years: 85.1), insomnia (age 50–59 years: 28.3 points vs age 70 + years: 19.2), and pain (age 40–49 years: 26.6 points vs age 60–69 years: 17.6 points); see Table 2.

In an additional analysis comparing participants above and below 60 years of age, participants ≥ 60 years old had better scores across all QLQ-C30 domains, including summary score, except physical functioning. The greatest mean differences were in emotional functioning (+ 8.7 points), insomnia (− 7.3 points), financial impact (− 6.5 points), social functioning (+ 5.8 points), and fatigue (− 5.8 points).

In women, by comparing age groups against the overall mean for women we found the five largest differences for: insomnia + 7.1 points (women aged 50–59 years), emotional functioning + 7.0 (women aged > 70 years), financial problems + 6.3 points (women aged 40–49 years), physical functioning − 5.9 points (women aged > 70 years), and pain + 5.7 (women aged 40–49 years). In men, the comparison of the age-group specific mean against the overall mean in men showed the five largest differences for: emotional functioning + 10.3 points, insomnia − 9.9 points, pain − 8.3 points, fatigue − 7.7 points (all in men aged > 70 years), and appetite loss + 6.6 points (men aged 18–39 years).

### Normative data by sex and age, and health condition

In the total sample, the largest differences between participants with and without health conditions were found for pain (30.6 points vs 10.1), global QOL (59.1 vs 79.3), and fatigue (30.3 vs 13.5). In men, the largest differences were observed for global QOL (61.2 vs 80.0), pain (28.4 vs 10.6), and role functioning (79.8 vs 94.2). In women, the largest differences were found for pain (32.6 vs 9.6) and global QOL (57.2 vs 78.6). All of these differences were in favour of participants without health conditions. For further details please see Table 5.

### Regression models for prediction of normative scores

To predict scores for each of the QLQ-C30 scales for an individual or a group, we developed regression models based on age, sex (0 = men, 1 = women), and health condition (0 = none, 1 = one or more). Details on the regression models are given in Supplementary Table S1.

The regression model uses years above 18 as the age variable (i.e. participant age minus 18). To give an example, for a female participant aged 50 years, and suffering from one or more health condition(s), the predicted score for Physical Functioning can be obtained via the following equation:

Physical Functioning (predicted) = 86.085 + sex * 2.514 + (age-18) * 0.529 + (age-18)^2^ * − 0.006 + sex * (age-18) * − 0.003 + health condition* − 11.426.

Physical Functioning (predicted) = 86.085 + 1 (female) * 2.514 + (50–18) * 0.529 + (50–18)^2^ * − 0.006 + 1 * (50–18) * − 0.003 + 1 (one or more health conditions) * − 11.426 = 87.861.

## Discussion

In this article, we have reported a detailed analysis of normative data for the EORTC QLQ-C30 in the general Spanish population. While we observed age- and sex-specific differences, the most important aspect with a substantial negative impact on all EORTC QLQ-C30 domains was the presence of a health condition. Scores in the QLQ-C30 for the overall sample were generally high, in line with the scores from the international study’s global sample [30]. Comparing the results from this analysis against the global sample published previously [14], differences between Spanish data and the global sample were trivial or small. Regarding summary score, Spain ranked 6^th^ among the 13 European countries analysed in the international study.

Fayers [35] has suggested possible reasons for these differences between countries, including health habits and cultural effects: communities may perceive their HRQOL differently due to variations in expectations. Other reasons could involve selection bias or differences in the interview systems [22], although this is not likely in the overall sample as the selection process was standardised across the different countries.

Our EORTC QLQ-C30 scores were aligned with those in the EORTC Reference Values manual for the general population [12]. Further, similar to our results, small differences by sex for emotional functioning and fatigue [14] were also found in the main general population study [30], other studies performed in Europe [1, 6, 17–19, 23, 26], and various other countries [25, 36]. Contrary to ours, however, most of those studies found differences in various HRQOL domains. Differences by sex in various countries have been considered to be related to health and lifestyle differences [5].

Our HRQOL results are in keeping with an Australian study that showed that older adults have higher overall HRQOL (highest scores for 11 QLQ-C30 domains) [36]. Contrary to our data, some other studies have reported substantially lower HRQOL in older participants [1, 4–6, 16, 23]; in others, age effects were weak [22, 26]. Nevertheless, some differences we found with sex and increasing age are aligned with results of the main general population study [30] and other QLQ-C30 studies [1, 6, 17] as well as the reference values study of the EuroQol-5D-5L for Spain [37].

Our higher item/scale scores for older adults could be related to people being better at adapting to situations as they age [38]. Also, older adults in Spain tend to have good health and life expectancies – among the highest in Europe: 86.1 years for women; 81.6 years for men [39]. Our results could also reflect the fact that patients > 80 years old were underrepresented in our sample (1.3% of participants), and a decline in HRQOL could be expected at this age [1, 5, 21].

Other QLQ-C30 studies have indicated declines in HRQOL in people with chronic health conditions [1, 5, 18, 21, 23]. Thus, the results of this and other studies highlight the importance of accounting for this variable in HRQOL studies of both cancer patients and the general population. In view of this finding, HRQOL of patients with cancer may be impacted more by comorbidities than by late-stage treatment effects [6, 23, 40].

As mentioned above, the use of normative data is only one way to facilitate interpretation of PRO scores. Unlike the concept of MIDs, which supports interpretations of PRO score differences between groups or time points, normative data is primarily applicable for interpreting cross-sectional data from individual patients or patient groups. In this regard, normative data provides a different perspective to thresholds (cut-offs), which categorise patients according to clinically relevant criteria [13]. Unlike using thresholds to guide interpretation, normative data maintains the level of information conveyed by scores. Normative data can even be integrated into the scoring of a PRO instrument itself, as is usually done by calculating T-scores [34].

A key consideration when using normative data is the selection of the reference population. We consider general population data the most appropriate comparator when interpreting PRO scores of cancer survivors, or when estimates of pre-disease levels of symptoms or functional health are required. For populations of patients undergoing active anti-cancer treatment, it may be more appropriate to rely on reference data from cancer patient populations that share essential disease and treatment characteristics.

This study has several limitations. It would have been interesting to include a higher number of people older than 80 to study the effect of aging on HRQOL in this group.

However, the authors of the main general population study [30] indicated obtaining a larger sample of this hard-to-reach group was outside the scope of their study as it would have substantially increased the budget for GfK which was not financially viable.

Also, our sample was relatively highly educated. This plus the lack of elderly people could be a consequence of conducting the surveys online. The prevalence of comorbidities such as cancer, COPD, or anxiety disorders in our sample compared well against Spanish national statistics, while the prevalence of diabetes and asthma was somewhat lower in our sample [42, 43]. The effect of comorbidity on HRQOL has been studied by organising participants into just two groups based on the presence/absence of comorbidities. It might be interesting to have a future study in which comorbidities can be studied in more detail.

## Conclusions

In conclusion, Spanish normative data presented in this article will enhance outcome interpretation in future studies, by providing benchmark data against which study findings from the EORTC QLQ-C30 could be compared. Our results highlight that age, sex and comorbid health conditions must be considered when comparing HRQOL data from the general population with that of cancer patients [24, 35]. Easier interpretation of scores from PRO instruments is key to fostering their wider use in clinical research and daily practice (Additional files: 1 and 2).

## Supplementary Information

**Additional file 1: Supplementary Table S1**. Regression models for the EORTC QLQ-C30 values in the general population of Spain.

**Additional file 2: Supplementary Table S2**. Participants’ demographic characteristics by age group.

## Data Availability

Data supporting our fndings can be made available upon request to the EORTC Qualiyt of Life Group.

## Abbreviations

EORTC: European Organisation for Research and Treatment of Cancer
QLG: Quality of Life Group
HRQOL: Health-related quality of life
MID: Minimal important differences
SD: Standard deviations

## References

[1] Mols F, Husson O, Oudejans M (2018). Reference data of the EORTC QLQ-C30 questionnaire: five consecutive annual assessments of approximately 2000 representative Dutch men and women. Acta Oncol.

[2] Büttner M, Zebralla V, Dietz A (2017). Quality of life measurements: any value for clinical practice?. Curr Treat Options Oncol.

[3] Jensen RE, Bjorner JBM (2019). Applying PRO reference values to communicate clinically relevant information at the point-of-care. Med Care.

[4] Feyers P, Bottomley A (2002). Quality of life research within the EORTC—the EORTC QLQ-C30. Eur J Cancer.

[5] Juul T, Petersen MA, Holzner B (2014). Danish population-based reference data for the EORTC QLQ-C30: associations with gender, age and morbidity. Qual Life Res.

[6] Velenik V, Secerov-Ermenc A (2017). Health-related Quality of Life Assessed by the EORTC QLQ-C30 Questionnaire in the General Slovenian Population. Radiol Oncol.

[7] Aaronson NK, Ahmedzai S, Bergman B (1993). The European Organization for Research and Treatment of Cancer QLQ-C30: a quality-of-life instrument for use in international clinical trials in oncology. J Natl Cancer Inst.

[8] Gnanasakthy A, Barrett A, Evans E (2019). A review of patient-reported outcomes labeling for oncology drugs approved by the FDA and the EMA (2012–2016). Value Health.

[9] Smith AB, Cocks K, Parry D, Taylor M (2014). Reporting of health-related quality of life (HRQOL) data in oncology trials: a comparison of the European Organization for Research and Treatment of Cancer Quality of Life (EORTC QLQ-C30) and the Functional Assessment of Cancer Therapy-General (FACT-G). Qual Life Res.

[10] Howell D, Molloy S, Wilkinson K (2015). Patient-reported outcomes in routine cancer clinical practice: a scoping review of use, impact on health outcomes, and implementation factors. Ann Oncol.

[11] Giesinger JM, Kieffer JM, Fayers PM (2016). Replication and validation of higher order models demonstrated that a summary score for the EORTC QLQ-C30 is robust. J Clin Epidemiol.

[12] Scott NW, Fayers PM, Aaronson NK, et al. EORTC QLQ-C30 reference values: Quality of Life Department, EORTC Headquarters; 2008. https://www.eortc.org/app/uploads/sites/2/2018/02/reference_values_manual2008.pdf. Accessed May 7 2020.

[13] Giesinger JM, Loth FLC, Aaronson NK (2020). Thresholds for clinical importance were established to improve interpretation of the EORTC QLQ-C30 in clinical practice and research. J Clin Epidemiol.

[14] Cocks K, King MT, Velikova G (2011). Evidence-based guidelines for determination of sample size and interpretation of the European Organisation for the Research and Treatment of Cancer Quality of Life Questionnaire Core 30. J Clin Oncol.

[15] Klee M, Groenvold M, Machin D (1997). Quality of life of Danish women: population-based norms for the EORTC QLQ-C30. Qual Life Res.

[16] Hjermstad M, Fayers P, Bjorda IK (1998). Health-related quality of life in the general Norwegian population assessed by the European Organization for Research and Treatment of Cancer Core Quality-of-Life Questionnaire: the QLQ=C30 (+ 3). J Clin oncol.

[17] Hjermstad MJ, Fayers PM, Bjordal K (1998). Using reference data on quality of life—the importance of adjusting for age and gender, exemplified by the EORTC QLQ-C30 (+3). Eur J Cancer.

[18] Michelson H, Bolund C, Nilsson B (2000). Health-related quality of life measured by the EORTC QLQ-C30–reference values from a large sample of Swedish population. Acta Oncol.

[19] Derogar M, van der Schaaf M, Lagergren P (2012). Reference values for the EORTC QLQ-C30 quality of life questionnaire in a random sample of the Swedish population. Acta Oncol.

[20] Schwarz R, Hinz A (2001). Reference data for the quality of life questionnaire EORTC QLQ-C30 in the general German population. Eur J Cancer.

[21] Waldmann A, Schubert D, Katalinic A (2013). Normative data of the EORTC QLQ-C30 for the German population: a population-based survey. PLoS ONE.

[22] Hinz A, Singer S, Brähler E (2014). European reference values for the quality of life questionnaire EORTC QLQ-C30: results of a German investigation and a summarizing analysis of six European general population normative studies. Acta Oncol.

[23] van de Poll-Franse LV, Mols F, Gundy CM (2011). Normative data for the EORTC QLQ-C30 and EORTC-sexuality items in the general Dutch population. Eur J Cancer.

[24] Yun YH, Kim SH, Lee KM (2007). Age, sex, and comorbidities were considered in comparing reference data for health-related quality of life in the general and cancer populations. J Clin Epidemiol.

[25] Finck C, Barradas S, Singer S (2012). Health-related quality of life in Colombia: reference values of the EORTC QLQ-C30. Eur J Cancer Care (Engl).

[26] Ficko SL, Pejsa V, Zadnik V (2019). Health-related quality of life in Croatian general population and multiple myeloma patients assessed by the EORTC QLQ-C30 and EORTC QLQ-MY20 questionnaires. Radiol Oncol.

[27] Tomás-Sábado J, Villavicencio-Chávez C, Monforte-Royo C (2015). What gives meaning in life to patients with advanced cancer? A comparison between Spanish, German, and Swiss Patients. J Pain Symptom Manage.

[28] Zbar AP, Gravitz A, Audisio RA (2012). Principles of surgical oncology in the elderly. Clin Geriatr Med.

[29] Di Maio M, Perrone F. Quality of Life in elderly patients with cancer. Health Qual Life Outcomes. 2003;44. 10.1186/1477-7525-1-44 PMC212193 14525617

[30] Nolte S, Liegl G, Petersen MA (2019). General population normative data for the EORTC QLQ-C30 health-related quality of life questionnaire based on 15,386 persons across 13 European countries, Canada and the Unites States. Eur J Cancer.

[31] United Nations (2017). World populatino prospects: key findings and advanced tables, 2017 revision.

[32] Arraras J, Arias F, Tejedor M (2002). The EORTC QLQ-C30 (version 3.0) Quality of Life questionnaire: validation study for Spain with head and neck cancer patients. Psychooncol.

[33] Fayers PM, Aaronson NK, Bjordal K, et al. EORTC QLQ-C30 scoring manual (3rd edn): the EORTC QLQ-C30. Brussels: European Organisation for Research and Treatment of Cancer; 2001.

[34] Liegl G, Petersen MA, Groenvold M (2019). Establishing the European Norm for the health-related quality of life domains of the computer-adaptive test EORTC CAT Core. Eur J Cancer.

[35] Fayers PM (2001). Interpreting quality of life data: population-based reference data for the EORTC QLQ-C30. Eur J Cancer.

[36] Mercieca-Bebber R, Costa DS, Norman R (2019). The EORTC Quality of Life Questionnaire for cancer patients (QLQ-C30): Australian general population reference values. Medical J Aust.

[37] Hernandez G, Garin O, Pardo Y (2018). Validity of the EQ-5D-5L and reference norms for the Spanish population. Qual Life Res.

[38] Idler E, Cartwright K (2018). What do we rate when we rate our health? Decomposing age-related contributions to self-rated health. J Health Soc Behav.

[39] OECD. Health status: life expectancy: organisation for economic cooperation and development; 2017. https://stats.oecd.org/index.aspx?queryid=30114#.

[40] Fosså SD, Hess SL, Dahl AA (2007). Stability of health-related quality of life in the Norwegian general population and impact of chronic morbidity in individuals with and without a cancer diagnosis. Acta Oncol.

[41] Kuijpers W, Giesinger JM, Zabernigg A (2016). Patients’ and health professionals’ understanding of and preferences for graphical presentation styles for individual-level EORTC QLQ-C30 scores. Qual Life Res.

[42] Ferlay J, Ervik M, Lam F, Colombet M, Mery L, Piñeros M, Znaor A, Soerjomataram I, Bray F. Global cancer observatory: cancer today. Lyon, France: international agency for research on cancer. https://gco.iarc.fr/today. Accessed 09 June 2021, 2020.

[43] Ministry of Health, Social Services and Equality (Spain), National Statistics Institute (Spain). Spain National Health Survey 2016–2017. Madrid, Spain: National Statistics Institute (Spain), 2018.

