# Supplementary material for: General Spanish population normative data analysis for the EORTC QLQ-C30 by sex, age, and health condition
Source: Health Qual Life Outcomes. 2021 Aug 30;19:208. doi: 10.1186/s12955-021-01820-x (PMC8404330; doi:10.1186/s12955-021-01820-x)
Supplement: Supplementary file 1 — Additional file 1: Supplementary Table S1. Regression models for the EORTC QLQ-C30 values in the general population of Spain. [file 12955_2021_1820_MOESM1_ESM.doc]

Supplementary Table S1: Regression models for the EORTC QLQ-C30 values in the general population of Spain

|  | Intercept | Sex* |  | Age* |  | Age* squared |  | Age*-by-sex |  | Health condition* |  |
| --- | --- | --- | --- | --- | --- | --- | --- | --- | --- | --- | --- |
|  |  | Coeff. | p-value | Coeff. | p-value | Coeff. | p-value | Coeff. | p-value | Coeff. | p-value |
| Physical Functioning | 86.085 | 2.514 | 0.128 | 0.529 | < 0.001 | -0.006 | 0.002 | -0.003 | 0.003 | -11.426 | < 0.001 |
| Role Functioning | 87.441 | 4.918 | 0.021 | 0.311 | 0.091 | -0.001 | 0.633 | -0.003 | 0.010 | -15.468 | < 0.001 |
| Emotional Functioning | 82.125 | -3.837 | 0.074 | -0.050 | 0.785 | 0.006 | 0.027 | < 0.001 | 0.675 | -13.318 | < 0.001 |
| Cognitive Functioning | 90.287 | -2.515 | 0.185 | 0.099 | 0.545 | < 0.001 | 0.871 | < 0.001 | 0.911 | -11.076 | < 0.001 |
| Social Functioning | 90.488 | -1.993 | 0.356 | 0.204 | 0.274 | 0.001 | 0.748 | -0.001 | 0.613 | -13.428 | < 0.001 |
| Global QOL | 74.594 | -1.245 | 0.543 | 0.188 | 0.287 | 0.001 | 0.711 | -0.001 | 0.184 | -20.956 | < 0.001 |
| Fatigue | 16.095 | 4.662 | 0.032 | -0.129 | 0.492 | -0.003 | 0.307 | 0.001 | 0.207 | 18.515 | < 0.001 |
| Nausea / Vomiting | 8.518 | -2.631 | 0.050 | -0.165 | 0.155 | -0.001 | 0.612 | 0.002 | 0.040 | 4.631 | < 0.001 |
| Pain | 14.982 | -1.894 | 0.406 | -0.044 | 0.824 | -0.004 | 0.112 | 0.004 | 0.001 | 20.433 | < 0.001 |
| Dyspnoea | 10.056 | -1.640 | 0.441 | -0.161 | 0.382 | < 0.001 | 0.892 | 0.001 | 0.445 | 13.191 | < 0.001 |
| Insomnia | 18.504 | 1.284 | 0.644 | 0.066 | 0.783 | -0.006 | 0.065 | 0.001 | 0.333 | 17.888 | < 0.001 |
| Appetite loss | 15.042 | -3.117 | 0.112 | -0.482 | 0.004 | 0.003 | 0.198 | 0.002 | 0.027 | 8.955 | < 0.001 |
| Constipation | 13.075 | -0.460 | 0.850 | -0.174 | 0.407 | 0.001 | 0.812 | 0.002 | 0.161 | 9.048 | < 0.001 |
| Diarrhoea | 11.243 | -3.387 | 0.057 | -0.142 | 0.354 | -0.002 | 0.442 | 0.002 | 0.065 | 6.123 | < 0.001 |
| Financial Problems | 6.674 | 0.167 | 0.935 | -0.023 | 0.897 | -0.003 | 0.223 | <0.001 | 0.911 | 10.989 | < 0.001 |
| Summary Score | 86.839 | 0.482 | 0.727 | 0.179 | 0.133 | 0.001 | 0.587 | -0.002 | 0.021 | -12.577 | < 0.001 |
