# Supplementary material for: General Spanish population normative data analysis for the EORTC QLQ-C30 by sex, age, and health condition
Source: Health Qual Life Outcomes. 2021 Aug 30;19:208. doi: 10.1186/s12955-021-01820-x (PMC8404330; doi:10.1186/s12955-021-01820-x)
Supplement: Supplementary file 2 — Additional file 2: Supplementary Table S2. Participants’ demographic characteristics by age group. [file 12955_2021_1820_MOESM2_ESM.doc]

| **Supplementary Table S2: Participants’ demographic characteristics by age group (N = 1165)** |  |  |  |  |  |  |  |  |  |  |  |  |  |  |  |  |  |  |  |  |  |
| --- | --- | --- | --- | --- | --- | --- | --- | --- | --- | --- | --- | --- | --- | --- | --- | --- | --- | --- | --- | --- | --- |
|  |  |  |  |  |  |  |  |  |  |  |  |  |  |  |  |  |  |  |  |  |  |
|  |  | **18-39 years** |  | **18-39 years** |  | **40-49 years** |  | **40-49 years** |  | **50-59 years** |  | **50-59 years** |  | **60-69 years** |  | **60-69 years** |  | **>=70 years** |  | **>=70 years** |  |
|  |  | **Unweighted** |  | **Weighted** |  | **Unweighted** |  | **Weighted** |  | **Unweighted** |  | **Weighted** |  | **Unweighted** |  | **Weighted** |  | **Unweighted** |  | **Weighted** |  |
|  |  | **N** | **%** | **N** | **%** | **N** | **%** | **N** | **%** | **N** | **%** | **N** | **%** | **N** | **%** | **N** | **%** | **N** | **%** | **N** | **%** |
| **Sex N (%)** | **Male** | 107 | 51,2% | 206 | 50,6% | 105 | 49,3% | 115 | 50,7% | 111 | 50,2% | 98 | 49,7% | 192 | 63,0% | 70 | 47,9% | 117 | 53,9% | 78 | 41,3% |
|  | **Female** | 102 | 48,8% | 201 | 49,4% | 108 | 50,7% | 112 | 49,3% | 110 | 49,8% | 99 | 50,3% | 113 | 37,0% | 76 | 52,1% | 100 | 46,1% | 111 | 58,7% |
| **Education N (%)** | **Less than compulsory education** | 2 | 1,0% | 5 | 1,2% | 1 | 0,5% | 1 | 0,4% | 0 | 0,0% | 0 | 0,0% | 3 | 1,0% | 2 | 1,4% | 8 | 3,7% | 8 | 4,3% |
|  | **Compulsory school** | 10 | 4,8% | 19 | 4,7% | 12 | 5,7% | 13 | 5,8% | 15 | 6,8% | 13 | 6,6% | 24 | 7,9% | 12 | 8,2% | 22 | 10,2% | 21 | 11,2% |
|  | **Some post-compulsory school** | 14 | 6,7% | 26 | 6,4% | 21 | 10,0% | 22 | 9,8% | 24 | 10,9% | 21 | 10,7% | 39 | 12,8% | 18 | 12,3% | 34 | 15,8% | 30 | 16,0% |
|  | **Post-compulsory below university** | 46 | 22,1% | 85 | 21,0% | 73 | 34,8% | 78 | 34,8% | 79 | 35,7% | 70 | 35,7% | 103 | 33,8% | 50 | 34,2% | 59 | 27,4% | 50 | 26,6% |
|  | **University degree (Bachelor)** | 79 | 38,0% | 161 | 39,9% | 65 | 31,0% | 69 | 30,8% | 66 | 29,9% | 59 | 30,1% | 93 | 30,5% | 43 | 29,5% | 71 | 33,0% | 61 | 32,4% |
|  | **Postgraduate Degree** | 57 | 27,4% | 108 | 26,7% | 38 | 18,1% | 41 | 18,3% | 37 | 16,7% | 33 | 16,8% | 43 | 14,1% | 21 | 14,4% | 21 | 9,8% | 18 | 9,6% |
|  | **Prefer not to answer** | 1 |  | 3 |  | 3 |  | 3 |  |  |  |  |  |  |  |  |  | 2 |  | 2 |  |
| **Marital status N (%)** | **Single/not in steady relationship** | 58 | 27,8% | 132 | 32,4% | 24 | 11,3% | 26 | 11,5% | 20 | 9,0% | 18 | 9,1% | 12 | 4,0% | 6 | 4,2% | 6 | 2,8% | 6 | 3,2% |
|  | **Married or in a steady relationship** | 148 | 70,8% | 270 | 66,3% | 171 | 80,7% | 182 | 80,5% | 167 | 75,6% | 149 | 75,6% | 217 | 71,9% | 99 | 69,2% | 151 | 69,6% | 124 | 65,6% |
|  | **Separated/divorced/widowed** | 3 | 1,4% | 5 | 1,2% | 17 | 8,0% | 18 | 8,0% | 34 | 15,4% | 30 | 15,2% | 73 | 24,2% | 38 | 26,6% | 60 | 27,6% | 59 | 31,2% |
|  | **Prefer not to answer** |  |  |  |  | 1 |  | 1 |  |  |  |  |  | 3 |  | 2 |  |  |  |  |  |
| **Employment status N (%)** | **Full-time employed** | 239 | 59,3% | 132 | 63,5% | 143 | 63,0% | 133 | 62,4% | 93 | 47,7% | 105 | 47,7% | 31 | 21,4% | 66 | 21,6% | 1 | 0,5% | 1 | 0,5% |
|  | **Part-time employed** | 50 | 12,4% | 25 | 12,0% | 22 | 9,7% | 21 | 9,9% | 22 | 11,3% | 24 | 10,9% | 8 | 5,5% | 14 | 4,6% | 2 | 1,1% | 3 | 1,4% |
|  | **Homemaker** | 9 | 2,2% | 5 | 2,4% | 16 | 7,0% | 15 | 7,0% | 23 | 11,8% | 26 | 11,8% | 14 | 9,7% | 21 | 6,9% | 23 | 12,2% | 21 | 9,7% |
|  | **Student** | 37 | 9,2% | 13 | 6,3% | 0 | 0,0% | 0 | 0,0% | 1 | 0,5% | 1 | 0,5% | 0 | 0,0% | 0 | 0,0% | 0 | 0,0% | 0 | 0,0% |
|  | **Unemployed** | 46 | 11,4% | 23 | 11,1% | 23 | 10,1% | 22 | 10,3% | 25 | 12,8% | 28 | 12,7% | 18 | 12,4% | 36 | 11,8% | 0 | 0,0% | 0 | 0,0% |
|  | **Retired** | 8 | 2,0% | 3 | 1,4% | 5 | 2,2% | 5 | 2,3% | 5 | 2,6% | 6 | 2,7% | 67 | 46,2% | 150 | 49,2% | 160 | 84,7% | 188 | 86,6% |
|  | **Self-employed** | 7 | 1,7% | 4 | 1,9% | 14 | 6,2% | 13 | 6,1% | 20 | 10,3% | 23 | 10,5% | 6 | 4,1% | 16 | 5,2% | 2 | 1,1% | 3 | 1,4% |
|  | **Other** | 7 | 1,7% | 3 | 1,4% | 4 | 1,8% | 4 | 1,9% | 6 | 3,1% | 7 | 3,2% | 1 | 0,7% | 2 | 0,7% | 1 | 0,5% | 1 | 0,5% |
|  | **Prefer not to answer** | 1 |  | 4 |  |  |  |  |  | 1 |  | 1 | 300,0% |  |  |  |  |  |  |  |  |
| **Comorbidity N (%)** | **None** | 97 | 48,7% | 201 | 52,5% | 70 | 34,1% | 74 | 33,9% | 74 | 34,7% | 66 | 34,7% | 105 | 35,6% | 49 | 34,8% | 45 | 21,2% | 38 | 20,5% |
|  | **One or more** | 102 | 51,3% | 182 | 47,5% | 135 | 65,9% | 144 | 66,1% | 139 | 65,3% | 124 | 65,3% | 190 | 64,4% | 92 | 65,2% | 167 | 78,8% | 147 | 79,5% |
|  | **Chronic Pain** | 36 | 18,1% | 59 | 15,4% | 56 | 27,3% | 60 | 27,5% | 60 | 28,2% | 53 | 27,9% | 58 | 19,7% | 28 | 19,9% | 42 | 19,8% | 39 | 21,1% |
|  | **Heart**  **Disease** | 2 | 1,0% | 3 | 0,8% | 5 | 2,4% | 5 | 2,3% | 8 | 3,8% | 7 | 3,7% | 16 | 5,4% | 6 | 4,3% | 24 | 11,3% | 20 | 10,8% |
|  | **Cancer** | 1 | 0,5% | 1 | 0,3% | 2 | 1,0% | 2 | 0,9% | 8 | 3,8% | 7 | 3,7% | 8 | 2,7% | 4 | 2,8% | 12 | 5,7% | 11 | 5,9% |
|  | **Depression** | 23 | 11,6% | 41 | 10,7% | 23 | 11,2% | 24 | 11,0% | 23 | 10,8% | 20 | 10,5% | 24 | 8,1% | 11 | 7,8% | 17 | 8,0% | 16 | 8,6% |
|  | **COPD** | 2 | 1,0% | 5 | 1,3% | 3 | 1,5% | 3 | 1,4% | 6 | 2,8% | 4 | 2,1% | 20 | 6,8% | 9 | 6,4% | 16 | 7,5% | 12 | 6,5% |
|  | **Arthritis** | 6 | 3,0% | 15 | 3,9% | 18 | 8,8% | 19 | 8,7% | 17 | 8,0% | 15 | 7,9% | 28 | 9,5% | 15 | 10,6% | 34 | 16,0% | 32 | 17,3% |
|  | **Diabetes** | 13 | 6,5% | 25 | 6,5% | 20 | 9,8% | 22 | 10,1% | 16 | 7,5% | 14 | 7,4% | 44 | 14,9% | 20 | 14,2% | 42 | 19,8% | 32 | 17,3% |
|  | **Asthma** | 24 | 12,1% | 42 | 11,0% | 15 | 7,3% | 16 | 7,3% | 7 | 3,3% | 6 | 3,2% | 5 | 1,7% | 2 | 1,4% | 8 | 3,8% | 8 | 4,3% |
|  | **Anxiety**  **disorder** | 22 | 11,1% | 38 | 9,9% | 22 | 10,7% | 22 | 10,1% | 21 | 9,9% | 19 | 10,0% | 20 | 6,8% | 9 | 6,4% | 12 | 5,7% | 12 | 6,5% |
|  | **Obesity** | 20 | 10,1% | 36 | 9,4% | 33 | 16,1% | 36 | 16,5% | 29 | 13,6% | 25 | 13,2% | 33 | 11,2% | 17 | 12,1% | 33 | 15,6% | 28 | 15,1% |
|  | **Drug/alcohol**  **disorder** | 3 | 1,5% | 5 | 1,3% | 1 | 0,5% | 1 | 0,5% | 0 | 0,0% | 0 | 0,0% | 0 | 0,0% | 0 | 0,0% | 0 | 0,0% | 0 | 0,0% |
|  | **Other** | 18 | 9,0% | 33 | 8,6% | 29 | 14,1% | 31 | 14,2% | 44 | 20,7% | 39 | 20,5% | 64 | 21,7% | 29 | 20,6% | 53 | 25,0% | 46 | 24,9% |
|  | **Prefer not to answer** | 9 |  | 21 |  | 8 |  | 9 |  | 8 |  | 7 |  | 6 |  | 3 |  | 4 |  | 3 |  |
|  | **Missing** | 1 |  | 3 |  | 0 |  | 0 |  | 0 |  | 0 |  | 4 |  | 2 |  | 1 |  | 1 |  |
